# Role of Hepatic Deposited Immunoglobulin G in the Pathogenesis of Liver Damage in Systemic Lupus Erythematosus

**DOI:** 10.3389/fimmu.2018.01457

**Published:** 2018-06-25

**Authors:** Xiang Fang, Muhammad Haidar Zaman, Xuanxuan Guo, Huimin Ding, Changhao Xie, Xiaojun Zhang, Guo-Min Deng

**Affiliations:** ^1^Key Laboratory of Antibody Technology, National Health and Family Planning Commission, Nanjing Medical University, Nanjing, China; ^2^First affiliated Hospital of Nanjing Medical University, Nanjing, China; ^3^State Key Laboratory of Reproductive Medicine, Nanjing Medical University, Nanjing, China

**Keywords:** systemic lupus erythematosus, hepatitis, immunoglobulin G deposition, innate immune cells, spleen tyrosine kinase

## Abstract

The onset of hepatic disorders in patients with systemic lupus erythematosus (SLE) is frequent; however, the etiology and liver pathogenesis of SLE remain unknown. In the present study, the role of hepatic deposited immunoglobulin G (IgG) in SLE-derived liver damage was investigated. From a retrospective analysis of the medical records of 404 patients with lupus and from experimental studies on mice models, we found that liver dysfunction is common in SLE and liver damage with IgG deposition spontaneously develops in lupus-prone mice. Liver injury was recreated in mice by injecting IgG from lupus serum intrahepatically. The inflammation intensity in the liver decreased with IgG depletion and the lupus IgG-induced liver inflammation in FcγRIII-deficient mice was comparatively low; while, inflammation was increased in FcγRIIb-deficient mice. Macrophages, Kupffer cells, natural killer cells, and their products, but not lymphocytes, are required for the initiation of SLE-associated liver inflammation. Blocking IgG signaling using a spleen tyrosine kinase (Syk) inhibitor suppressed the liver damage. Our findings provided evidence of spontaneously established liver damage in SLE. They also suggested that hepatic-deposited lupus IgG is an important pathological factor in the development of liver injury and that hepatic inflammation is regulated by the Syk signaling pathway. Thus, Syk inhibition might promote the development of a therapeutic strategy to control liver damage in patients with SLE.

## Introduction

Systemic lupus erythematosus (SLE) is a chronic autoimmune disease that possesses the feature of elevated autoantibodies and associated damage in the tissues of multiple organs (1–3). SLE-derived liver failure is rare, and true liver disease triggered by SLE is a debatable issue; however, 25–50% of patients with SLE present with alterations in liver function tests (LFTs). Therefore, we must explore the cellular and molecular mechanisms involved in the SLE-derived liver pathogenesis (4–7).

The relationship between SLE and variation in LFTs might be caused by three possibilities: (1) an overlap of SLE with another autoimmune liver disease; (2) comorbidity of SLE and a non-autoimmune hepatopathy; or (3) the existence of liver parenchymal injury that only relates to SLE (lupus hepatitis) (8, 9). The first category, which occurs with higher incidence, is the overlapping of liver injury ascribed by autoimmune mechanisms, such as autoimmune hepatitis (AIH) or primary biliary cirrhosis, with hepatopathies triggered by SLE (5, 10). Frequently, patients with SLE have comorbidities with many non-autoimmune hepatic diseases, such as drug-derived hepatic injury, viral or infectious hepatitis, and thrombotic liver diseases (11–14). The prevalence of lupus hepatitis lies between 6.1 and 24.5% (14–16); however, there are currently no diagnostic gold-standard criteria and specific treatment for lupus hepatitis and the diagnosis is mainly rule out on the bases of other liver diseases. Thus, it is important to understand the pathogenesis and features of lupus hepatitis.

Evidence has been found for the involvement of autoantibodies in the pathogenesis of certain autoimmune diseases, including SLE. The course of action in SLE-derived tissue injuries is related to autoantibody production, the formation of immune complexes, and their deposition (17). Our team (18) and another study (19) showed that humans lupus serum and lupus-prone mice serum could induce damage to certain organs. In addition, conventional T (20) and B (21) cells also perform a critical function in the development of organ lesions in SLE. However, studies on lupus serum-derived skin inflammation demonstrated that inflamed skin tissue attracts lymphocytes to the inflamed site, where they participates in lesion chronicity but are not necessary for the development of tissue pathology (18). As an organ with predominantly innate immunity, liver tissue comprises immune cells like macrophage/Kupffer cells, natural killer (NK) cells, and other innate immune cells. These defending cells protect the liver against pathogenic infection, liver injury, inflammation, and fibrosis (22). However, evidence suggests that these innate immune cells also contribute to the pathogenesis of hepatitis (23). Several studies demonstrated that the elevated pro-inflammatory cytokine and chemokine level, which are markers of chronic inflammation, increase disease progression, and facilitate the recruitment of neutrophils, monocytes, and macrophages (24, 25). Despite these lines of evidence, the underlying mechanism of the pathogenesis of lupus-derived liver damage remains unknown.

The current study aimed to determine the possible role of immunoglobulin G (IgG) in SLE-derived liver pathogenesis. We analyzed the clinical information in the medical records of 91 patients with liver dysfunction (LD) selected from among 404 patients with SLE and investigated the pathogenesis of liver damage in SLE using animal models. We confirmed that liver damage develops spontaneously in lupus disease. We also found that hepatic deposited lupus IgG is an important pathological factor in the development of liver injury in SLE. Based on our findings, we suggested that spleen tyrosine kinase (Syk) inhibitors could be used as prognostic drugs for the occurrence, severity, and anti-inflammation status in patients with SLE-related hepatitis. Our findings contribute to future research on the development of a therapeutic strategy to prevent or treat liver damage in SLE.

## Materials and Methods

### Patients

Data from 404 inpatients fulfilling four or more revised American College of Rheumatology (ACR) criteria (26) for the categorization of SLE in the First Affiliated Hospital of Nanjing Medical University between December 2012 and December 2013 were reviewed to retrospectively analyze the clinical features of lupus hepatitis. The data gathered included patient biophysical information (age, gender, and duration of disease), clinical manifestations, and various hematological parameters [including alteration in the level of alanine aminotransferase (ALT), aspartate aminotransferase (AST), γ-glutamyl transferase (GGT), alkaline phosphomonoesterase (ALP), total bilirubin (TBILI), and direct bilirubin (DBILI)] of every patient.

### Mice and Sera Samples

C57BL/6 (B6), ICR, and BALB/c mice; and Sprague Dawley (SD) rats were obtained from the Animal Center of Nanjing Medical University. *RAG-1*^−/−^ (J002216), B6.MRL-*Fas^lpr^*/J (B6 lpr) (J000482), and *TLR4*^−/−^ (J003752) mice were purchased from Model Animal Research Center of Nanjing University. *Fc*γ*RIIb*^−/−^ (002848), *Fc*γ*RIII*^−/−^ (003171), TNF-α^−/−^ (005540), and MRL/MpJ-*Fas^lpr^*/J (MRL-*lpr*) (000485) mice were purchased from the Jackson Laboratories (USA). Pathogen-free and temperature stabilized environment with *ad libitum* access to food and water were provided to all mice at the Animal Core Facility of Nanjing Medical University. All animal experiments were approved by the Nanjing Medical University Institutional Animal Care and Use Committee (IACUC-14030140). All mice used in this study were female with 6–8 weeks of age, unless otherwise indicated.

Mice sera were collected from MRL/*lpr*, B6 lpr, and normal C57BL/6 mice. While human lupus serum and human normal serum were kindly provided by the First Affiliated Hospital of Bengbu Medical College and the First Affiliated Hospital of Nanjing Medical University. From the provided lupus serum samples from patients, nineteen patients were selected who satisfied ≥4 of the 11 revised criteria of the ACR for the classification of SLE. The SLE disease-activity index for all of these patients had scores ranging from 0 to 18. Along with lupus serum, sera from four normal individuals were also used as controls.

At serum collection time, all participants expressed no sign or symptom of any other infection except SLE. Informed consent was received from all patients and healthy donors under the Nanjing Medical University Review Board-approved protocol. Unless otherwise indicated, all experimental procedures were performed using serum from patients with SLE. The characteristics of all patients with SLE and the control group are summarized in Table S1 in Supplementary Material.

Some of the lupus sera were utilized to extract lupus IgG, using Protein G Agarose beads (MILLIPORE, USA), following the manufacturer’s standard protocol (27). The purity of the extracted IgG was confirmed using electrophoresis.

Recombined murine IFN-γ and TNF-α were purchased from Pepro Tech. Syk inhibitor R406 (sc-364595A, LOT# D2814) was purchased from Santa Cruz Biotechnology (Dallas, TX, USA).

### Western Blotting Analysis

For cytokine expression studies, liver tissues were lysed in radio immunoprecipitation assay (RIPA) buffer. Total lysates (20 µg) were subjected to 12% sodium dodecylsulfate-polyacrylamide gel electrophoresis and then shifted to polyvinylidene fluoride membrane (Millipore). TNF-α and IFN-γ were detected using primary antibodies (Abs): anti-TNF-α (Abcam, ab6671) and anti-IFN-γ (Bioworld technology, BS3486) at 1/1,000 dilution. Goat anti-mouse β-actin was used as a loading control. To detect intracellular signaling proteins, cell were lysed using RIPA buffer with a protease inhibitor cocktail and phosphatase inhibitor (Roche). The following Abs were used (from CST unless otherwise indicated): anti-Syk antibody (Cat. 2712), anti-phospho-Syk (p-Syk) antibody (Cat. 2717), anti-IκBα antibody (Cat. 4812), anti-p-IκBα antibody (Cat. 2859), anti-NF-κB p65 antibody (Cat. 4764), and anti-p-NF-κB p65 antibody (Cat. 3031). All Abs were used at 1/1,000 dilution. The appropriate horseradish peroxidase-conjugated Related-IgG (Abbkine, USA) were used as the secondary antibody for all samples. Visualization of immunoreactivity proteins was performed using an ECL Western Blotting Analysis System, following the manufacturer’s guideline (GE Healthcare).

### Histology, Immunohistochemistry (IHC), Immunofluorescence, and Microscopy

For histological analysis, liver tissue was fixed using 10% buffered formalin. After fixation, the tissues were dehydrated in ethanol, embedded in paraffin, and stained with Hematoxylin and eosin (H&E). All slides were marked and then interpreted thoroughly for information. Liver inflammation severity was scored from 0 to 5 (28): grade 0 = normal, grade 1 = mild inflammation around parts of portal tracts, grades 2–3 = different amounts of infiltrating inflammatory cells around the portal tracts, grade 4 = ballooning degeneration of hepatocytes, and grade 5 = extensive destruction of liver cells. For IHC analysis, the following mouse Abs (from Abcam, unless otherwise indicated) were used: anti-mouse IgG antibody (clone RM 104, ab190475), anti-CD3 antibody (clone SP7, ab16669), anti-F4/80 antibody (clone CI: A3-1, ab6640), anti-CD11c antibody (clone N418, ab33483), and anti-CD20 antibody (Santa Cruz, clone M-20, sc-7735). After treatment with primary Abs, the slides were incubated with biotinylated secondary Abs. All sections were counterstained with Mayer’s hematoxylin. For immunofluorescence staining, frozen tissues were treated with anti-Human-IgG Abs (Abcam, clone EPR4421, ab109489) followed by incubation with fluorescein isothiocyanate (FITC)-conjugated secondary Abs and then counterstained with 2-(4-amidinophenyl)-1H-indole-6-carboxamidine (DAPI). Images were captured using a Zeiss LSM700 confocal microscope and ZEN software (Carl Zeiss, Thornwood, NY, USA).

### Intrahepatic Injection Protocol and Cell Depletion

A single intrahepatic injection of lupus serum or IgG was administrated to induce acute liver injury. Mice and rats were anesthetized intraperitoneally (i.p.) with pentobarbital sodium (Merck, USA). Following disinfecting the abdomen, a 1-cm-long surgical incision was made at a mid-abdominal site and then serum or IgG was injected intrahepatically in mice and rats, using a disposable insulin syringe, and the surgical incision was sutured.

Kupffer cells were depleted using 200 µL clodronate-loaded liposomes administered by intravenous injection in experimental/test mice (29) 1 day before lupus IgG administration, while 200 µL of empty liposome-phosphate-buffered saline (PBS) were injected to control mice instead of clodronate-loaded liposomes. To selectively deplete NK cells, 20 µL of anti-asialo GM1 (ASGM1) antibody (Wako, Japan) per mouse were injected intraperitoneally, 24 h before IgG treatment (30). The eradication of Kupffer and NK cells was confirmed using flow cytometry (Figure S4 in Supplementary Material).

### Analysis of Liver Transaminase Activities

For blood collection, a sterilized glass capillary tube was used to puncture the orbital venous plexus of mice. Thirty minutes after blood collection, the serum from blood was separated and collected by centrifugation at 2,000 g for 10 min at 4°C. The collected sera were preserved at −20°C until analysis. At specific time points after IgG administration, liver injuries were assessed by measuring ALT and AST transaminase levels using a kit (Jiancheng Bio., China).

### Isolation of Liver Mononuclear Cells (MNCs) and Flow Cytometric Analysis

For the isolation of liver MNCs (31), liver tissues were passed through a 70-µm cell strainer (FALCON, USA), followed by resuspension in 40% and then 70% Percoll. The cells were then centrifuged at 750 × *g* for 30 minutes at room temperature, after which the MNCs were isolated from the interphase.

For phenotyping and functional analysis of liver MNCs, multi-parametric flow cytometry was used. The monoclonal antibodies (mAbs) used for liver phenotyping were: an allophycocyanin-conjugated anti-CD3e antibody (eBioscience, clone 145-2C11, 17-0031), a phycoerythrin-conjugated anti-NK1.1 antibody (BD Pharmingen, clone PK136, 557391), and a FITC-conjugated anti-CD69 antibody (BD Pharmingen, clone H1.2F3, 557392). All Abs were used at 1/200 dilution. Cells were treated with mAbs for 30 min at 4°C at saturated concentrations of mAbs. All samples were run on a flow cytometer (BD FACS calibur) and analyzed using the FlowJo software (Tree Star).

To detect apoptosis quantitatively and qualitatively, the liver MNCs were isolated from lupus-prone mice. We incubated 100 µL of 1 × 10^6^ cells/mL of isolated MNCs for 15 min at 25°C in the dark with 5 µL of Annexin V-FITC and 5 µL of propidium iodide (BD Bioscience, 556420). The samples were then analyzed on a FACS Calibur flow cytometer and the percentage of positively stained cells was calculated using FlowJo.

### Isolation and Culture of Bone Marrow-Derived Macrophages

As a source of macrophage colony-stimulating factor, we prepare and use L929-cell conditioned medium (L929 CM) according to the method of Hosoe et al. (32). Roswell Park Memorial Institute-1640 media plus 10% fetal bovine serum were used to incubate the L929 cells for 5–7 days. Thereafter, leaving the confluent monolayer cells at the bottom of the culture plate, the supernatant without cell were collected and filtered by passing through a 0.22-µm filter. The filtered supernatant was stored at −20°C until use.

To obtain bone marrow-derived macrophages (BMDM), C57BL/6 mice were sacrificed through cervical dislocation and then, using cool serum-free media, the bone marrow cells (BMCs) were flushed from femoral shafts. To get rid of adherent macrophages, BMC single cell suspension washing was performed by incubating BMCs in a culture flask with serum-free media for 4 h. Macrophages-free BMCs were then cultured in the isolated culture media from the L929 cells (10% v/v). After incubation, viable cells were washed with warm medium to remove non-adherent cells, leaving the adherent cells as the obtained BMDMs. The adherent cells were then cultured in new complete culture medium.

### Analysis of Apoptosis Using TUNEL Assay

To further determine the type and distribution of apoptotic cells, the paraffin embedded tissue sections were deparaffinized and the terminal deoxynucleotidyl transferase-mediated dUTP-biotin nick end labeling (TUNEL) assay was performed using an apoptosis detection kit by Roche, Germany, according to the manufacturer’s guideline.

### Statistical Analysis

GraphPad PRISM software was used to analyze the data statistically, with *P*-values < 0.05 considered as significant, applying two-tailed Student’s *t*-test. All data are presented as the mean ± SD. According to the experimental parameter, all experiments were repeated at least three times and each time the sample size in each group was 5–7.

## Results

### Features of Liver Damage in Lupus Disease

**Figure 1:**
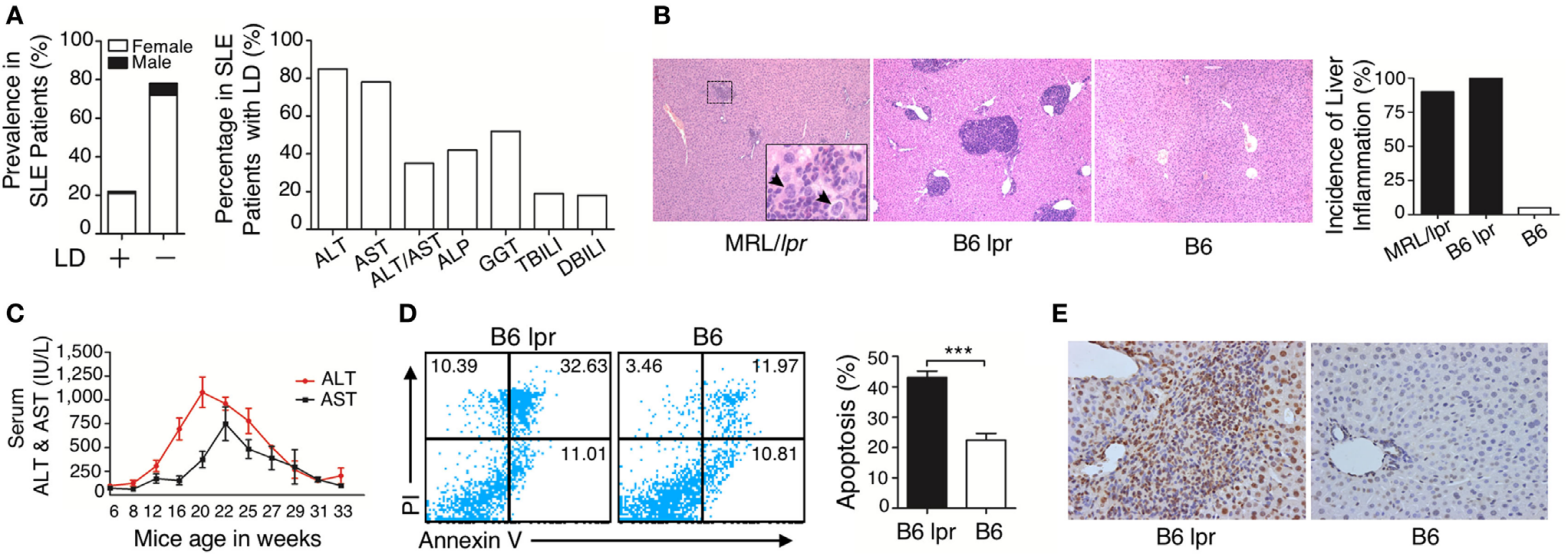
Characterization of liver damage in lupus disease. **(A)** Clinical information in the cohort of 404 systemic lupus erythematosus (SLE) inpatients was reviewed and liver dysfunction (LD) was detected in 91 out of 404 SLE inpatients, while the other 313 SLE inpatients without LD served as the control group (normal liver function group). Serum biochemical markers of LD in 91 SLE patients are shown. **(B)** Histopathological examination of the liver and incidence of hepatic inflammation from lupus-prone mice MRL/*lpr* (*n* = 7, 22 weeks), B6 lpr (*n* = 10, 30 weeks), and B6 mice (*n* = 10, 30 weeks). Infiltration of inflammatory cells around the portal vein in these two mice models with different genetic backgrounds. The arrow indicates ballooning degeneration of hepatocytes that was observed in ~5% of the portal area. Original magnification, 100×. **(C)** Serum levels of liver enzymes in B6.MRL/lpr (B6 lpr) mice at various ages. ALT, alanine aminotransferase; AST, aspartate aminotransferase (*n* = 10). **(D)** Analysis of apoptosis of liver cells from B6 lpr mice (30 weeks) and age-matched control B6 mice. ***P < 0.001 (the result is representative of three independent experiments, *n* = 4 mice per group/experiment.) **(E)** Representative photomicrographs of the TUNEL *in situ* assay of liver sections of B6 lpr mice (30 weeks) with hematoxylin as a counter-stain. Original magnification, 400×.

To investigate the pathogenesis of liver damage in patients with SLE, we analyzed the medical record of 404 patients with confirmed SLE disease. Among the patients with SLE, different serum biochemical parameters related to liver function and dysfunction were observed, including ALT, AST, ALT/AST, ALP, GGT, TBILI, and DBILI. Based on these data, especially the ALT and AST levels, the intensity of liver damage and LD were observed. Summarizing the data, among the 404 patients with lupus, 91 (22.5%; 82 females and 9 males; median age, 38 years old) patients were observed to have LD, while the other 313 SLE inpatients (77.5%) lacked LD (290 females and 23 males; median age, 39 years old) (Figure 1A). Age of the most patients with LD ranged from 19 to 54 years (Figure S1 in Supplementary Material). To confirm whether liver damage is caused by lupus disease, we used lupus-prone mice that could spontaneously develop lupus-like clinical and histopathological features. Histopathological examination demonstrated that many inflammatory cells were present around the portal areas of the liver in lupus-prone mice (Figure 1B). We also investigated changes in serum biochemical parameters of liver function in lupus-prone mice and found that serum ALT and AST peaked at the age of 20–22 weeks and then decreased as the disease progressed (Figure 1C). Apoptosis is one of the fundamental mechanisms involved in the pathogenesis of liver disease (33); therefore, we assessed apoptosis of the liver using flow cytometry (Figure 1D) and TUNEL staining (Figure 1E). The results showed that liver apoptosis existed in lupus-prone mice. We did not observe liver inflammation and apoptosis in lupus-prone mice at 5 weeks of age. Since chronic liver damage frequently leads to fibrosis (33, 34), we also assessed and confirmed fibrosis development in the livers of lupus-prone mice (Xiang Fang, unpublished data). These findings suggested that liver damage develops spontaneously in lupus-prone mice.

### Liver Inflammation Is Triggered by Hepatic Deposited Lupus IgG

**Figure 2:**
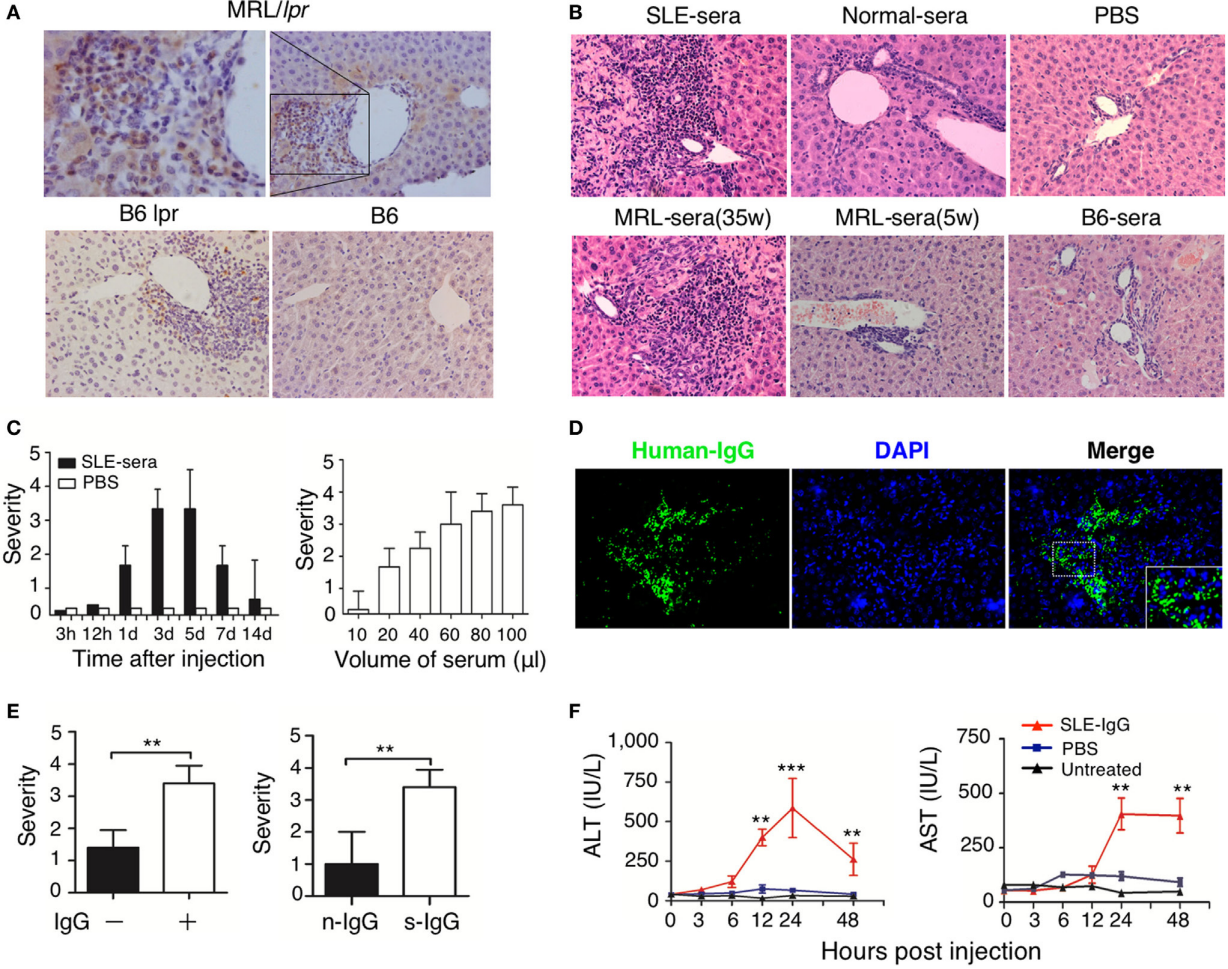
Hepatic deposited lupus serum immunoglobulin G (IgG) induced liver inflammation in normal mice. **(A)** Immunohistochemical staining detected IgG deposition in the liver of MRL/lpr mice (25 weeks), B6 lpr mice (30 weeks), and C57BL/6 mice (30 weeks). Shown is a representative image of IgG deposition around the inflammatory portal area of a lupus-prone mice liver. Original magnification, 200× **(B)** Representative histopathologic photomicrograph of liver sections of C57BL/6 mice sacrificed 3 days after intrahepatic injection of serum (100 µL) from a lupus patient with liver disease, healthy control, lupus-prone MRL/*lpr* mice (35 and 5 weeka), and normal C57BL/6 mice or phosphate-buffered saline (PBS). H&E staining; original magnification, 200× (the result is representative of three independent experiments, *n* = 5 mice per group/experiment). **(C)** Kinetics of liver inflammation induced by intrahepatic injection of serum (100 µL) from a lupus patient with liver disease (left panel) and the severity of hepatic lesions triggered by various volumes of systemic lupus erythematosus (SLE)-serum 3 days after intrahepatic injection (right panel) in normal C57BL/6 mice (the result is representative of three independent experiments, *n* = 7 mice per group/experiment). **(D)** Intrahepatic IgG deposition on liver sections was examined by immunofluorescence 3 days after SLE-serum injection. Original magnification, 100×. **(E)** The severity of liver inflammation 3 days after intrahepatic injection of 100 µL SLE-serum with or without IgG depletion and 200 µg IgG from patients with SLE (s-IgG) or normal healthy individuals (n-IgG). **p < 0.01 (the result is representative of three independent experiments, *n* = 5 mice per group/experiment.) **(F)** Values of serum alanine aminotransferase and AST at various time points after intrahepatic injection of SLE-IgG or PBS in C57BL/6 mice. **p < 0.01, ***p < 0.001 (the result is representative of three independent experiments, *n* = 5 mice per group/experiment.).

Immunoglobulin G deposition is an important factor that mediates tissue inflammation in SLE (1, 2); therefore, we investigated IgG deposition in the liver of lupus-prone mice. We found a large amount of IgG deposition around the portal area, especially in the regions of liver damage (Figure S2 in Supplementary Material; Figure 2A). This finding suggests that IgG might exert an important role in the development of liver damage in mice with lupus disease. To confirm whether deposited lupus IgG induces liver inflammation, we constructed C57BL/6 mice models with hepatic deposition of lupus IgG by injecting serum from lupus patients and from lupus-prone mice intrahepatically. For the control group, we injected serum from healthy humans and mice. As non-serological control, we also injected PBS intrahepaticaly into mice. The results showed that serum from SLE patients induced liver inflammation, while serum from healthy human and PBS did not induce inflammation (Figure 2B, upper panels). The same results were obtained when mouse serum was injected. The serum isolated from MRL/*lpr* mice with lupus induced inflammation, but serum collected during premorbid period from normal C57BL/6 mice did not (Figure 2B, lower panels). Liver inflammation was also not caused by lipopolysaccharide (LPS) contamination of the serum, because SLE-serum induced similar liver inflammation in mice with or without TLR-4 deficiency (Figure S5A in Supplementary Material). These data indicate that lupus serum causes liver inflammation.

Furthermore, liver inflammation was observed just 1 day after injecting lupus serum intrahepaticaly, and reached a peak on the third day (Figure 2C, left panel). The liver damage was characterized by infiltration of inflammatory cells into the portal area. It was also noted that the volume of the injected serum controlled the severity of liver inflammation (Figure 2C, right panel). Intrahepaticaly injected lupus serums-derived liver inflammation was also checked in other mouse strains, including BALB/c and ICR mice, along with SD rats (Figure S5C in Supplementary Material). All rodent strains showed the same results as those observed using C57BL/6 mice. However, we did not observe any liver inflammation following intraperitoneal and intravenous injection of the same volume of lupus serum (Figure S5B in Supplementary Material) in these mice. These findings supported the view that lupus IgG deposited in the liver causes the liver inflammation.

To further determine the role of IgG in lupus serum-derived hepatic destruction, we performed further experiments. IgG deposition was observed using Immunofluorescent staining of C57BL/6 mice livers injected intrahepaticaly with lupus serum (Figure 2D). Furthermore, we isolated IgG-free (depleted) lupus serum using protein G agarose beads (18). IgG separation and depletion was confirmed by electrophoresis (Figure S3 in Supplementary Material). We found a significant decrease in the liver inflammation of mice injected with lupus sera having no or depleted IgG and IgG-containing complexes, as compared with normal lupus sera without IgG depletion (Figure 2E, left panel). We also intrahepatically injected IgG or IgG-containing complexes isolated from lupus serum of mice to C57BL/6 mice and observed that lupus IgG could directly induce liver inflammation. It was also confirmed that using the same dose of IgG isolated from lupus serum could cause more severe liver damage compared with that caused by IgG isolated from healthy subject (Figure 2E, right panel). Changes caused by liver inflammation to the serum ALT and AST levels were also observed in mice after lupus IgG injection (Figure 2F). In addition, we observed an accumulation of apoptotic hepatocytes around the inflamed sites in the liver following lupus IgG stimulation (Figure S6 in Supplementary Material).

Thus, our study indicated that IgG or IgG-containing complexes are major contributors to the development of liver inflammation induced by lupus serum.

### Cellular and Cytokine Requirement for the Development of Liver Inflammation Induced by Hepatic Deposited Lupus IgG

**Figure 3:**
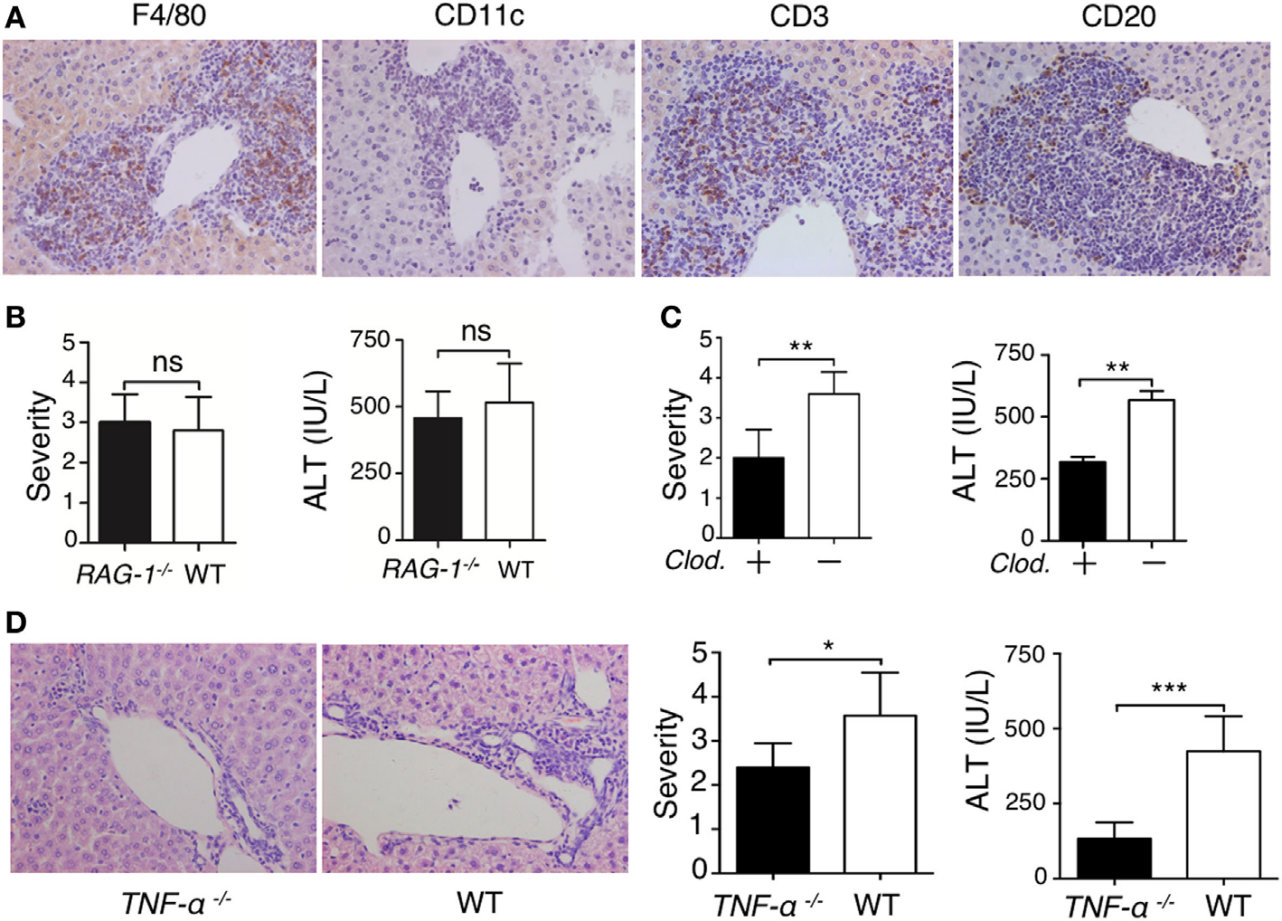
Liver inflammation triggered by intrahepatic deposited lupus immunoglobulin G (IgG) depends on macrophages and TNF-α. **(A)** Immunohistochemistry detected Kupffer cells (F4/80+), dendritic cells (CD11c+), T cells (CD3+), and B cells (CD20+) in liver sections from lupus-prone mice. The results were from three independent experiments. Original magnification, 200× **(B)** Severity of liver inflammation and serum alanine aminotransferase (ALT) level 48 h after intrahepatic injection of systemic lupus erythematosus (SLE)-IgG (200 µg) in RAG-1^–/–^ and wild-type mice (the result is representative of three independent experiments, *n* = 5 mice per group/experiment). **(C)** Severity of liver inflammation and serum ALT level 48 h after intrahepatic injection of SLE-IgG (200 µg) in macrophage-depleted mice with clodronate liposomes (*Clod*.)-pretreated and control mice. **p < 0.01 (the result is representative of three independent experiments, *n* = 5 mice per group/experiment). **(D)** Representative histopathologic photomicrograph showing the severity of liver inflammation and serum ALT level 48 h after intrahepatic injection of SLE IgG in wild-type and TNF-α deficient mice. *p < 0.05, ***p < 0.001 (the result is representative of three independent experiments, *n* = 5 mice per group/experiment).

To understand the mechanism of lupus IgG-induced liver damage, we investigated the role of immune cells involved in lupus hepatitis. First, we identified the types of inflammatory cells that infiltrated into the liver of lupus-prone mice. Immunohistochemical staining demonstrated that large numbers of F4/80−, CD3−, and CD20+ cells were present at the site of liver lesions (Figure 3A). To explore the role of lymphocytes in the progression of lupus IgG-induced liver damage, we used RAG-1-deficient mice that lack mature T and B cells (18). We found that the severity of liver inflammation and changes in the liver enzymes induced by lupus IgG were not significantly decreased in RAG-1-deficient mice compared with those in wild-type mice (Figure 3B). Macrophages are pivotal in several murine models of hepatitis (35, 36); therefore, to understand the role of monocytes/macrophages in the development of lupus IgG-induced liver lesions, we used macrophage-depleted mice that were pretreated with *clodronate liposomes* (29) and found that the severity of the liver lesions and liver enzymes levels induced by lupus IgG were significantly reduced in the macrophage-depleted mice compared with that in normal mice (Figure 3C). These data suggested that liver macrophages (Kupffer cells), but not lymphocytes, are important in the development of lupus-IgG-derived hepatic inflammation.

TNF-α is a pro-inflammatory cytokine that is mainly produced by monocytes/macrophages (18, 37, 38); thus, the role of TNF-α in the development of lupus-associated liver lesions was investigated. We injected lupus-IgG intrahepatically into TNF-α-deficient mice (*TNF-*α^−/−^) and found that the liver lesions were significantly reduced in the *TNF-*α^−/−^ mice (Figure 3D; Figure S13A in Supplementary Material). This finding suggested that lupus IgG-mediated liver inflammation might depend on Kupffer cell and TNF-α. The results demonstrated that macrophage and pro-inflammatory TNF-α are required for the development of hepatic inflammation induced by lupus IgG.

### Synergic Effect of Macrophage and NK Cells on Hepatic Apoptosis Induced by Lupus IgG

**Figure 4:**
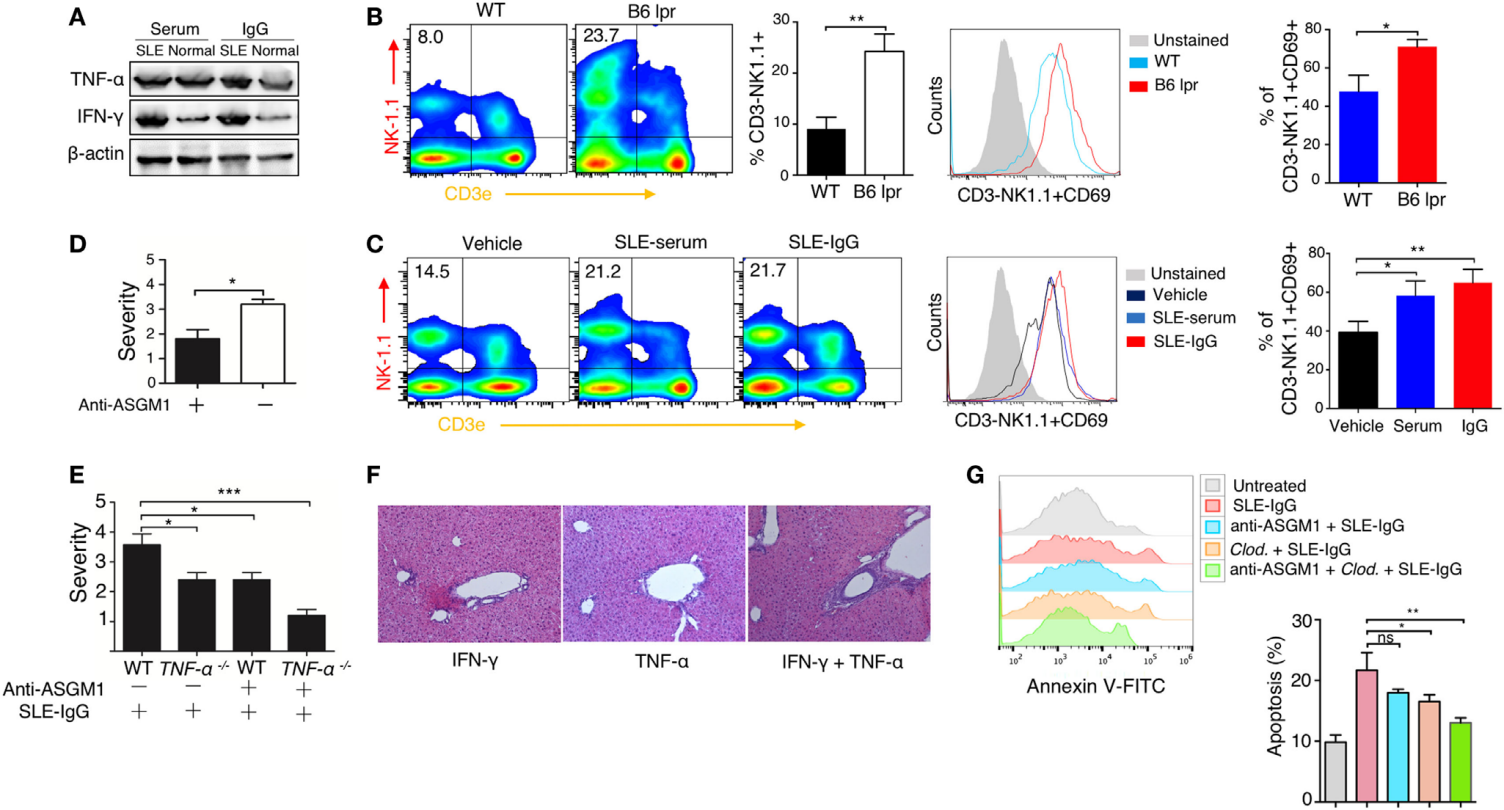
Synergistic effect of macrophage and natural killer (NK) cells on hepatic apoptosis induced by lupus immunoglobulin G (IgG). **(A)** Western blot showing the levels of TNF-α and IFN-γ in the liver from C57BL/6 mice with intrahepatic injection of the same dose of systemic lupus erythematosus (SLE) and normal serum, SLE, and normal IgG. **(B)** Flow cytometry analysis of NK cell activation in the liver from B6 lpr (25 w) and age-matched control mice using anti-NK1.1, anti-CD3e and anti CD69 antibodies. The result is representative of three independent experiments, *n* = 6 mice per group/experiment. **(C)** Flow cytometry analysis of NK cell activation in the liver from C57BL/6 mice with intrahepatic injection of SLE-serum (treated for 72 h), SLE-IgG (treated for 48 h), or phosphate-buffered saline (treated 48 h) using anti-NK1.1, anti-CD3e and anti CD69 antibodies (the result is representative of three independent experiments, *n* = 5 mice per group/experiment). **(D)** Severity of liver inflammation 48 h after intrahepatic injection of SLE-IgG in C57BL/6 mice with or without NK depletion using anti-ASGM1 antibody treatment. *p < 0.05 (the result is representative of three independent experiments, *n* = 5 mice per group/experiment.) **(E)** Severity of liver inflammation 48 h after intrahepatic injection of SLE-IgG (200 µg) in wild type and TNF-α deficient mice with or without NK cell depletion, respectively (***p < 0.001, *p < 0.05 the result is representative of three independent experiments, *n* = 5 mice per group/experiment). **(F)** Representative images of liver inflammation triggered by intrahepatic injection of recombined murine IFN-γ (500 ng/mouse) and/or TNF-α (200 ng/mouse) by HE staining. Original manifestation, 100× (the result is representative of three independent experiments, *n* = 7 mice per group/experiment). **(G)** Analysis of apoptotic hepatocytes 48 h after intrahepatic injection of SLE-IgG (200 μg/mouse) in mice with macrophage or NK cell depletion (the results are representative of three independent experiments, *n* = 4 mice per group/experiment).

We then examined the intrahepatic production of a series of pro-inflammatory cytokines. Immunoblotting revealed that TNF-α and IFN-γ levels were enhanced following serum or IgG treatment (Figure 4A; Figure S11 in Supplementary Material). There are abundant NK and NKT cells present in the liver (39, 40); therefore, we sought to determine the role of these cells in the development of lupus hepatitis. We found higher numbers of NK cells and their increased activation in the livers of lupus-prone mice (Figure 4B) and in the liver of those mice treated with intrahepatic injection of lupus serum or lupus IgG, compared with those in the controls (Figure 4C). To further assess the requirement for NK cells in liver inflammation induced by lupus IgG, we treat mice with anti-ASGM1 Abs, which selectively engage and deactivate NK cells (41). The results showed that the severity of liver inflammation induced by lupus IgG was attenuated in mice treated with anti-ASGM1 Abs compared with that in mice not treated with anti-ASGM1 Abs (Figure 4D). We also confirmed that in lupus IgG-induced liver lesions, NK cells produce IFN-γ (Figure S7 in Supplementary Material). To further explore the roles of TNF-α and IFN-γ in the development of liver injury after lupus IgG intrahepatic treatment, we inject lupus-IgG into TNF-α-deficient mice (TNF-α^−/−^), with or without NK cell-depletion, and found that the liver lesions were significantly reduced in both TNF-α^−/−^ mice and NK-depleted mice. In addition, we also found that depletion of NK cells in TNF-α^−/−^ mice almost completely abolished the hepatic inflammation following lupus IgG administration (Figure 4E). From the effects of injecting a sensitizing individual dose of recombined murine TNF-α, IFN-γ and their co-injection, we found that when combined, they had a distinct proinflammatory effect compared with the effect of the injection of either factor alone (Figure 4F; Figure S10 in Supplementary Material). We further determined the role of TNF-α and IFN-γ in hepatocyte apoptosis induced by lupus-IgG *in vivo*. We found that hepatic apoptosis was markedly attenuated in Kupffer cells-depleted or NK cell-depleted mice, while hepatic apoptosis was remarkably decreased in mice depleted for both Kupffer cell and NK cell (Figure 4G; Figure S12 in Supplementary Material). These data suggested that TNF-α and IFN-γ have a synergic effect on hepatic apoptosis induced by lupus IgG.

### Blocking IgG Signaling Suppressed Liver Inflammation Induced by Hepatic Deposited Lupus IgG

**Figure 5:**
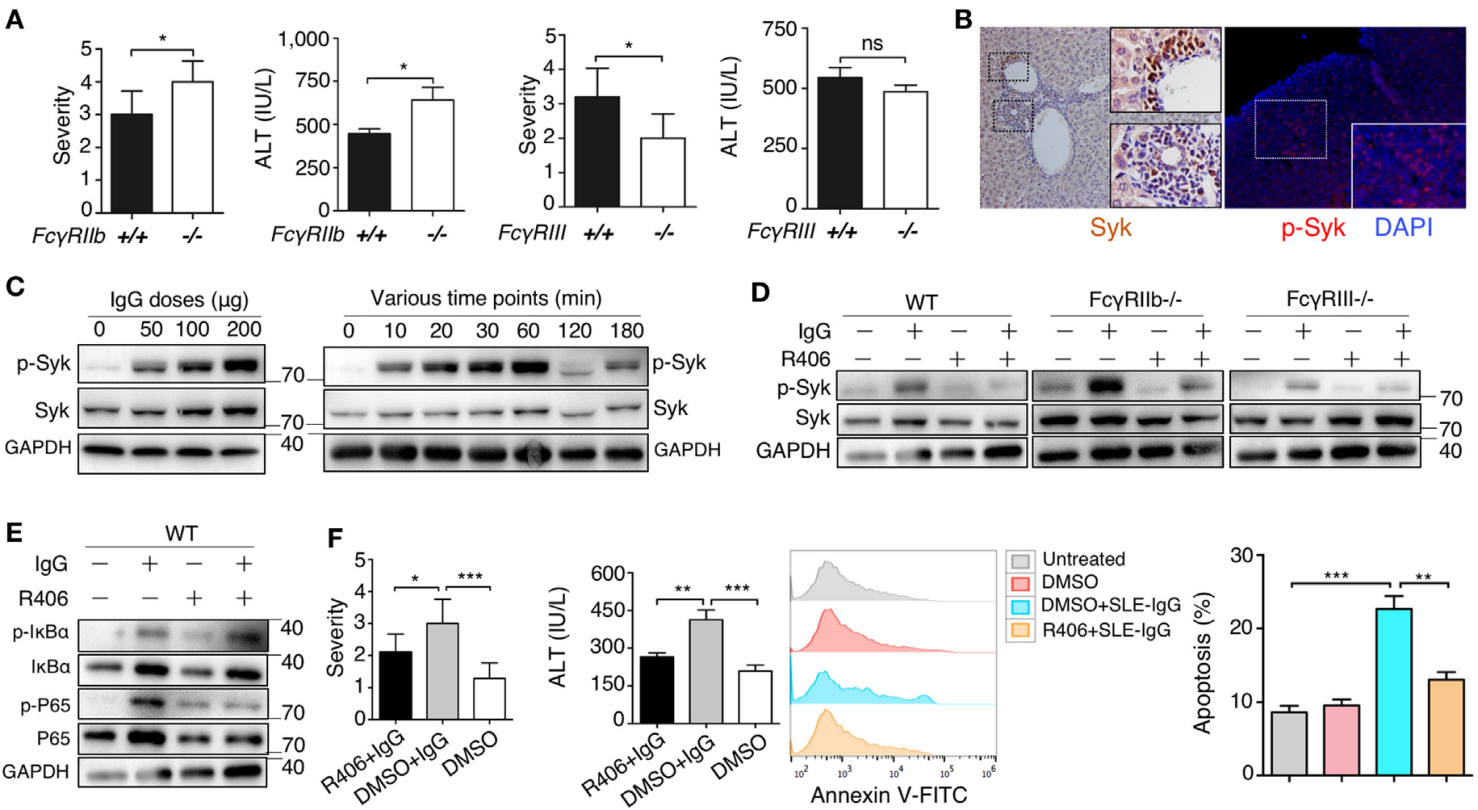
Spleen tyrosine kinase (Syk) inhibitor suppressed liver inflammation induced by deposited lupus immunoglobulin G (IgG). **(A)** Severity of liver inflammation and serum alanine aminotransferase (ALT) level 48 h after intrahepatic injection of systemic lupus erythematosus (SLE)-IgG in mice with FcγRIIb-deficient (FcγRIIb^−/−^) and FcγRIII-deficient (FcγRIII^−/−^). *p < 0.05 (the result is representative of three independent experiments, *n* = 6 mice per group/experiment). **(B)** Representative image of Syk and p-Syk expression in inflamed liver sections from lupus-prone mice (aged 29 weeks) by immunohistochemistry and immunofluorescence, respectively. **(C)** Western Blot detected phosphorylation of Syk (p-Syk) or Syk in BMDMs stimulated by various doses of SLE-IgG for 30 min or stimulated by SLE-IgG (100 µg/ml) for the indicated time points as shown. BMDMs (2 × 10^6^ cells) were isolated from wild-type (WT) mice. The results were from three independent experiments. **(D)** Western blot analyzing p-Syk or Syk in BMDMs stimulated by 100 µg/ml SLE-IgG in presence or absence of Syk inhibitor R406 (2 µM) for 30 min. BMDMs (2 × 10^6^ cells) were isolated from WT or FcγR^−/−^ mice. The results are from three independent experiments. **(E)** WT-derived BMDMs (2 × 10^6^ cells) were stimulated with 100 µg/ml SLE-IgG in the presence or absence of Syk inhibitor R406 (2 µM) for 30 min. Cell lysates were subjected to immunoblotting analysis to detect the designated proteins or their phosphorylated cognates. **(F)** The severity of liver inflammation, serum ALT level, and analysis of apoptotic hepatocytes in C57BL/6 mice treated with or without Syk inhibitor R406 (10 mg/kg body) sacrificed 48 h after intrahepatic injection of SLE-IgG (200 µg) from a patient with lupus. *p < 0.05, **p < 0.01, ***p < 0.001 (the result is representative of three independent experiments, *n* = 7 mice per group/experiment).

To further understand the molecular mechanism underlying the effect of deposited IgG on hepatic inflammation, we used FcγRIIb-deficient and FcγRIII-deficient mice. We found that severity of liver inflammation induced by lupus IgG increased in FcγRIIb-deficient mice, but decreased in FcγRIII-deficient mice compared with that in wild-type mice (Figure 5A; Figures S13B,C in Supplementary Material). Syk exerts a key role in FcR signaling pathway; therefore, we determine the expression of Syk in the liver lesions. IHC showed high levels of Syk in the hepatic-infiltrating inflammatory cells of lupus-prone mice. We also found that activated Syk (p-Syk) levels were markedly increased in the inflammatory lesions (Figure 5B). To further understand whether Syk regulates liver inflammation induced by deposited lupus IgG, we stimulated bone marrow derived macrophages (BMDMs) with lupus IgG *in vitro*. We found that treatment with lupus IgG promoted Syk activation in BMDMs in a dose-dependent manner (Figure 5C, left panel). Phosphorylation of Syk (p-Syk) induced by lupus IgG was significantly increased within 1 h and then was subsequently degraded (Figure 5C, right panel). We also found that a Syk inhibitor suppressed the increase in p-Syk induced by lupus IgG. Furthermore, deficiency of FcγRIIb could accelerate Syk activation, while deficiency of FcγRIII attenuated Syk activation, induced by lupus IgG, compared with that in the wild-type (Figure 5D). We also examined the lupus-IgG induced intracellular IgG/Syk-NF-kB signaling pathway and found that blocking of Syk signaling results in the inhibition of IgG/Syk-NF-kB pathway (Figure 5E). Thus, the present study confirmed that Syk activation is required for lupus IgG-induced liver damage. We further determined whether Syk inhibition could suppress the liver inflammation induced by deposited lupus IgG. We found that treatment of mice with a Syk inhibitor led to a significant reduction in liver injury, as demonstrated by histopathology and alteration in serum ALT levels between Syk inhibitor plus lupus IgG treated group of C57BL/6 mice and the lupus IgG only treated group (Figure 5F). In addition, an *in vivo* experiment showed that Syk inhibitor treatment of mice significantly reduced the apoptosis of hepatocytes induced by deposited lupus IgG (Figure 5F). The data indicated that Syk plays an important role in liver inflammation induced by deposited lupus IgG.

## Discussion

Although abnormal liver function in patients with SLE has been well documented, the pathogenesis of liver injury in SLE is not clear. Our clinical study of patients with SLE and practical experiments on animals demonstrated that liver injury is established in SLE and that hepatic deposition of lupus IgG is an important pathological factor in the development of SLE-associated liver injury. Additionally, Kupffer cells, NK cells, and their products play a major role in liver injury regulated by Syk in SLE.

It is difficult to determine whether liver injury is only caused by lupus disease in patients with SLE because liver injury can be initiated by many other factors, including drugs, viral infection, steatosis, vascular thrombosis, and AIH (42). As discussed earlier, the 91 patients that participated in our study had LD, and we know that some drugs that are used to treat SLE could be associated with liver abnormalities, e.g., *Methotrexate, Cyclophosphamide*, and *Azathioprine* (14). Therefore, it is very important to clarify that although drug-induced liver toxicity was present in patients with SLE, the hepatic injury in all these patients was not derived only by drugs. To confirm this, we used the identical SLE model of lupus-prone mice with a history of no drug or medication use, to clearly confirm the hypothesis that liver damage can be caused by only lupus disease in SLE. Based on the histopathology data, liver damage developed in all experimental lupus-prone mice, which allowed us to conclude that lupus disease can truly mediate liver damage. Interestingly, serum enzymes measurements revealed that abnormal change in liver function in the MRL/lpr mice started at 12 weeks and peaked at 20–22 weeks, but gradually normalized as the disease progressed (Figure 1C). Based on quantitative liver function data and hepatic histopathology images of the lupus-prone mice, the serum enzymes measurements, and the diagnostic imaging results in lupus patients, we considered that clinical serum biochemical changes in liver function cannot fully match the hepatic pathologic conditions. Thus, we speculated that incidence of actual liver damage is higher than that reported in patients with SLE.

The animal model of deposited lupus IgG-induced liver inflammation through intrahepatic injection of lupus serum in normal mice is a useful tool to investigate the pathogenesis of liver damage in SLE. Liver inflammation in this model was not caused by LPS contamination of lupus serum or by the surgical procedure, but it was associated with the dose of the injected lupus serum. Immunohistochemical analysis revealed that mouse IgG was widely distributed around the normal hepatic portal area and abundant IgG was deposited within the inflamed site in the liver of active lupus mice. We postulated that IgG deposition occurs earlier than the development of inflammation. Based on this model, we determined that liver inflammation is related to hepatic IgG deposition.

There are several pieces of evidences to support the hypothesis that hepatic IgG deposition exerts a critical role in the development of liver damage in SLE. SLE is characterized with a high level of autoantibody in serum (1); tissue IgG deposition is associated with skin and kidney damage (18); intrahepatic injection of lupus IgG induced liver inflammation; and liver inflammation induced by lupus IgG was reduced in FcγRIII-deficient mice, but increased in FcγRIIb-deficient mice. More severe liver inflammation was observed in mice treated with intrahepatic injection of IgG isolated from lupus serum than in those mice that were intrahepaticly administrated with the same volume of IgG extracted from healthy serum. This result suggested that lupus IgG contains immune complexes that are not present in healthy serum, because patients with SLE have a large number of autoantigens and autoantibodies.

Although we showed that liver inflammation is related to the dose of lupus IgG, we were surprised that liver damage was not related to the type of autoantibody, because the damage to the brain and kidney was associated with anti-dsDNA Ab (42, 43). We found that intrahepatic injection of autoantibody-laden serum from patients with primary Sjogren’s syndrome and rheumatoid arthritis mediated a similar pathological reaction in mice (Figure S8A in Supplementary Material). Although the clinical data from patients with SLE indicated that anti-dsDNA Abs might be important contributors to hepatitis development and that certain autoantibodies are connected to distinct pathologies in patients with SLE (44), the injection of sera with higher titers of other Abs still induced similar liver inflammation (Figure S8B in Supplementary Material).

Liver IgG deposition is a major pathological factor that initiates liver inflammation; therefore, tissue resident immune cells play an important role in the development of liver damage in SLE. Our data demonstrated that liver inflammation induced by lupus IgG required both Kupffer cells and NK cells, but did not require lymphocytes from circulating blood. This finding is supported by recent reports that Kupffer cells are poised to initiate the innate immune response through reciprocal interactions with local NK cells when primed by pathogen-derived products (45). Monocytes are an important resource for dendritic cells (DCs) (46, 47), which are one of the major types of antigen presenting cells in the liver (22). Although the histochemical staining of 25W lupus prone mice showed very few DCs present in the inflamed hepatic site (Figure 3A), our previous study showed that lupus serum IgG promoted monocyte differentiation into DCs (18), which might result in an increase in systemic circulatory DCs.

TNF-α plays an important role in the liver damage induced by lupus IgG. Kupffer cells can produce a large amount of TNF-α (22), which can directly induce liver inflammation (41). Our data demonstrate that liver inflammation induced by lupus IgG was remarkably reduced in mice with Kupffer cell depletion and TNF-α deficiency. By contrast, lupus IgG enhanced the serum TNF-α level in normal mice, but not in macrophage-depleted mice (Figure S9 in Supplementary Material).

Our study also demonstrates that hepatic inflammation induced by lupus IgG was affected by both NK cells and Kupffer cells. These data indicate that crosstalk exists between NK cells and Kupffer cells during the induction of liver damage. It has been shown that an interaction between human NK cells and monocytes can occur through the production of IFN-γ and TNF-α, respectively (48). Our data also demonstrated that these two cytokines had a synergic effect on liver damage.

Our findings demonstrate that Syk is an important therapeutic target in liver damage induced by lupus IgG deposition. FcR signal transduction is critically dependent on immune-receptor tyrosine-based activation motifs, which are located in its cytoplasmic tail (49, 50). Syk binds these phosphorylated ITAM motifs and activates downstream signaling. We confirmed that active Syk initiated the signaling pathways of IKK/NF-κB (Figure 5E) and alleviated the inflammation following lupus IgG administration *in vivo* (Figure 5F).

In summary, our study demonstrated that the liver is an important target organ that is affected by SLE-induced hepatic damage, which is induced by IgG deposited in liver tissue. IgG deposition in the liver tissue of patients with SLE leads to the activation of Kupffer cells and NK cells, and facilitate the production of TNF-α and pro-inflammatory cytokines. In this study, we not only explored the mechanism of liver pathology in SLE, but also demonstrated that Syk is a therapeutic target to treat liver damage in SLE. The findings of this study will contribute to future research to develop a therapeutic strategy to prevent or treat SLE associated liver damage.

## Ethics Statement

This study was carried out in accordance with the recommendations of “IACUC of guidelines, Nanjing Medical University of Committee.” The protocol was approved by the “Nanjing Medical University of Committee.”

## Author Contributions

XF and G-MD conceived the research plan, designed experiments, analyzed the data, and wrote manuscript with the contribution from MZ. XF performed the majority of the experiments. MZ, XG, HD, CX, and XZ contributed data for the manuscript.

## Conflict of Interest Statement

The authors declare that the research was conducted in the absence of any commercial or financial relationships that could be construed as a potential conflict of interest.
