# Supplementary material for: Role of Hepatic Deposited Immunoglobulin G in the Pathogenesis of Liver Damage in Systemic Lupus Erythematosus
Source: Front Immunol. 2018 Jun 25;9:1457. doi: 10.3389/fimmu.2018.01457 (PMC6026631; doi:10.3389/fimmu.2018.01457)
Supplement: Supplementary file 1 [file data_sheet_2.PDF]

Supplementary Table: Participant's data

Table. The detailed information of all patients and healthy subjects that participated in the present study are summarized.

Sample	Liver enzymes (U/L)				Complements (g/L)		Immunoglobulin (g/L)			Autoantibody (+/-)			SLEDAI
	ALT	AST	ALP	GGT	C3	C4	IgG	IgA	IgM	ds-DNA	Ro52	P0	
SLE1	154	662	364	401	0.144	0.120	19.90	3.83	0.87	+	+	−	5
SLE2	19	28	52	18	0.429	0.040	18.60	2.04	0.89	+	−	+	6
SLE3	23	34	86	29	0.968	0.213	11.90	1.43	0.33	−	−	−	16
SLE4	20	16	47	21	0.603	0.127	14.60	2.45	1.15	−	−	+	2
SLE5	16	20	66	19	0.413	0.053	9.21	0.86	0.96	+	−	+	12
SLE6	18	20	61	24	0.376	<0.017	26.50	1.41	1.11	−	+	−	3
SLE7	26	18	88	15	0.584	0.139	13.60	3.62	1.24	−	−	−	4
SLE8	12	16	46	21	0.359	0.039	15.90	2.54	1.19	−	+	−	9
SLE9	40	78	59	21	0.388	0.101	12.10	2.87	1.10	+	−	−	15
SLE10	34	29	147	57	0.655	0.203	7.95	2.73	0.73	+	−	+	10
SLE11	188	182	434	1238	0.119	<0.017	16.4	2.47	1.04	+	+	+	18
SLE12	255	95	95	170	0.512	0.059	18.5	3.16	3.00	+	−	+	13
SLE13	70	107	83	57	0.47	0.107	15.3	3.47	1.99	+	−	+	6
SLE14	183	366	78	24	0.765	0.130	40.9	6.54	2.28	−	+	−	12
SLE15	54	129	49	16	0.415	<0.017	17.9	3.76	2.02	+	−	+	6
SLE16	50	47	77	36	0.687	0.171	36.5	6.68	2.65	−	+	−	10
SLE17	39	46	211	296	1.010	0.353	17.3	3.48	0.43	+	−	−	4
SLE18	177	84	96	34	0.830	0.291	12.1	2.18	0.30	+	+	+	11
SLE19	403	246	212	147	0.853	0.151	7.82	1.94	0.68	−	+	−	9

Positive (＋); Negative (－)

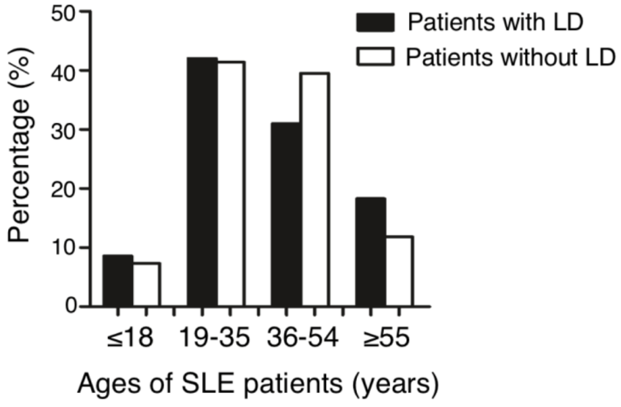

Fig. S1. The distribution of 91 selected SLE patients with LD, based on age and gender.

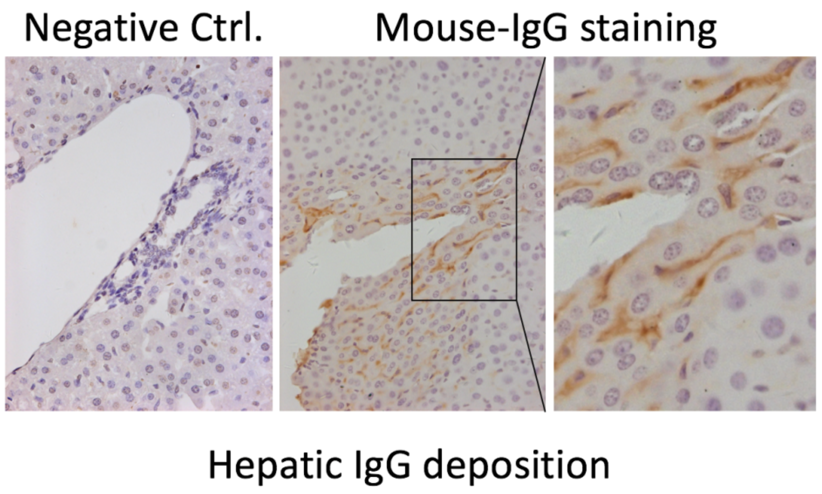

Fig. S2. Representative Immunohistochemical images of hepatic IgG deposition around the non-inflammatory portal area in the liver of MRL/lpr mice (25 weeks). Original magnification, ×400

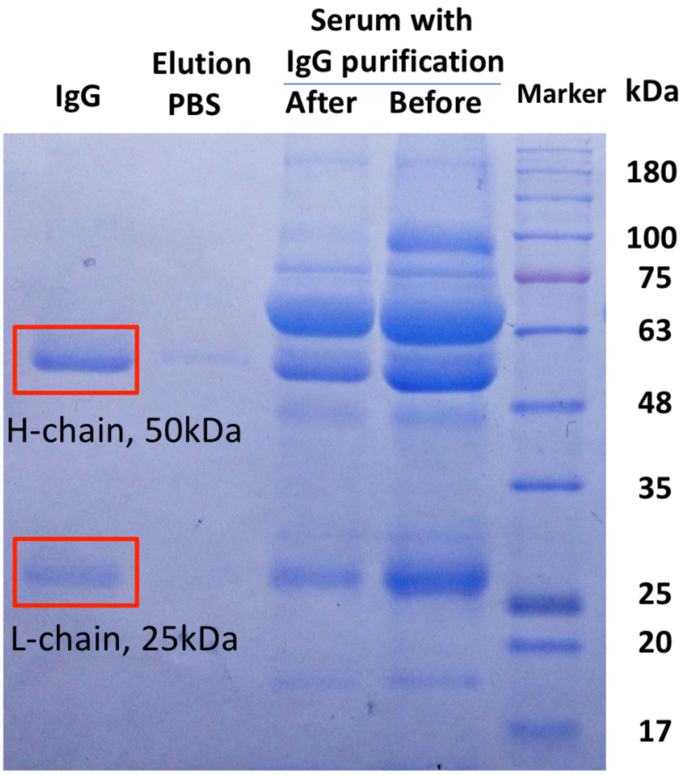

Fig. S3. SDS-PAGE analysis of IgG extracted from the blood of lupus patients. IgG separation and isolation from serum was done by immune-precipitation. Protein G agarose beads were used according to manufacturer's instruction to separate IgG from sera. Purity determination was performed using SDS-PAGE. Heavy (50kDa) and light (25kDa) chain were detected.

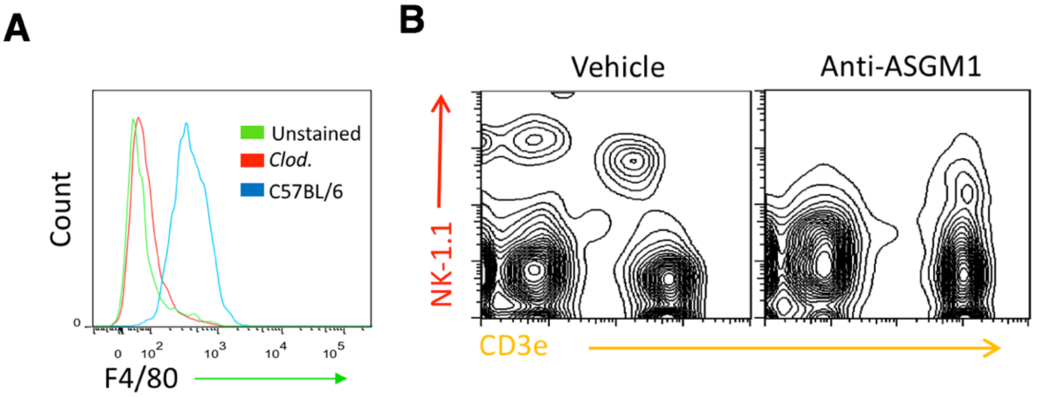

Fig. S4. The tendency of Kupffer and NK cells depletion.

(A) Liver mononuclear cells (MNCs) were isolated and analyzed using flow cytometry with an anti-F4/80 antibody and the result revealed that the clodronate (*Clod.*) *Liposomes* deplete the monocyte/macrophage population by 90%.

(B) C57BL/6 mice were administered with intraperitoneal injection of either 50 μL anti-asialo GM1 (Anti-ASGM1) or vehicle as a control. Flow cytometry analysis revealed that the population of NK 1.1 hepatic mononuclear cells from mice injected with a single dose of Anti-ASGM1 was reduced from 8.8% to 0.2%.

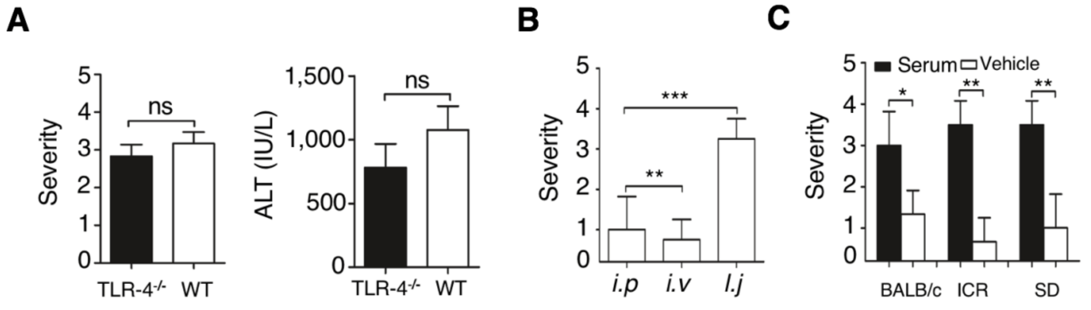

Fig. S5. Intrahepatic injection of lupus serum induced liver inflammation

(A) Toll like receptor-4-deficient ($TLR\text{-}4^{-/-}$) mice and wild-type were injected with SLE serum, and the severity of hepatic inflammation and serum alanine aminotransferase (ALT) concentration was assessed to determine the degree of liver injury. (The result is representative of three independent experiments, n= 6 mice per group/experiment.)

(B) Severity of liver inflammation in C57BL/6 sacrificed 3 d after intraperitoneal (i.p), intravenous (i.v) and intrahepatic (I.j) injection of serum (100 μL) from a lupus patient with liver disease. (The result is representative of three independent experiments, n= 7 mice per group/experiment.)

(C) Different strains of BALB/c mice, ICR mice and SD rats received PBS and sera from lupus patient and the histological score are shown. (The

result is representative of three independent experiments, n= 7 mice per group/experiment.) *p<0.05, **p<0.01, ***p<0.001

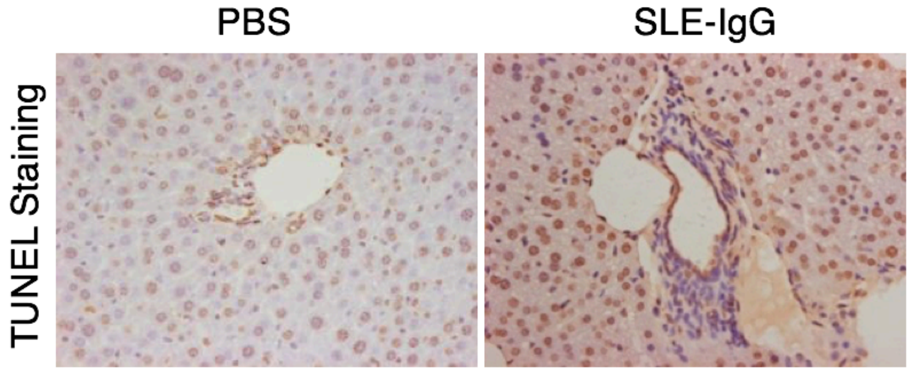

Fig. S6. Intrahepatic injection of SLE-IgG induced hepatic apoptosis.

SLE-IgG (200 μg/mouse) was injected intrahepatically into B6 mice. Apoptosis of liver cells was detected by an *in situ* TUNEL assay of liver sections at 48 h following IgG administration and then with haematoxylin as a counterstain. Original magnification, ×400

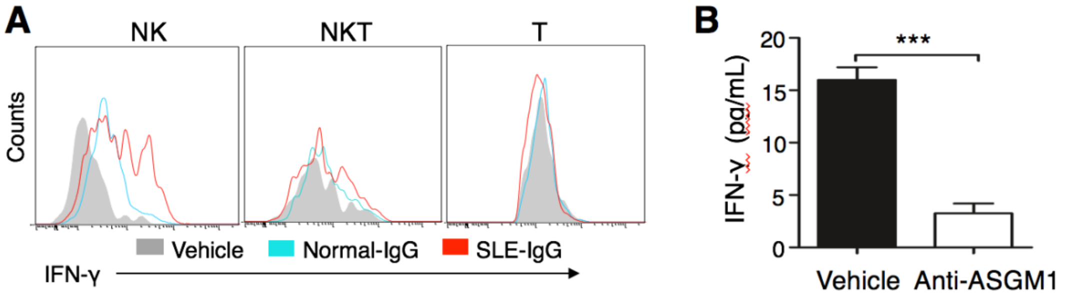

Fig. S7. NK cells produce IFN-γ after SLE-IgG intrahepatic deposition

(A) Flow cytometry of expression of IFN-γ on NK, NKT or T cells using anti-NK1.1, anti-CD3 and anti-IFN-γ antibodies in the liver 18h after intrahepatic injection of SLE IgG in C57BL/6 mice.

(B) Mice were pretreated with the anti-ASGM1 mAb or vehicle on two consecutive days before SLE-IgG injection; serum IFN-γ level was measured by ELISA 18h after intrahepatic injection of SLE IgG in mice with or without NK depletion. (The result is representative of three independent experiments, n= 6 mice per group/experiment.). ***p<0.001

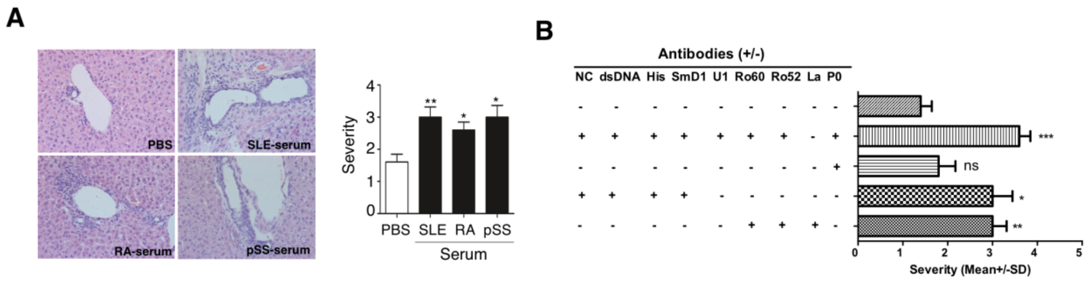

Fig. S8. Liver damage induced by autoantibody-laden serum intrahepatic deposition

(A) Histopathological characteristics of liver dysfunction in mice triggered by autoantibody-laden serum. These results reflect that there were no changes in liver inflammation in lupus-hepatitis. Original magnification, ×200

(B) Severity of liver sections of C57BL/6 mice sacrificed 3d after intrahepatic injection of serum (100 μL) from a lupus patient with or without the indicated antibodies. (The result is representative of three independent experiments, n= 6 mice per group/experiment); *p<0.05, **p<0.01, ***p<0.001

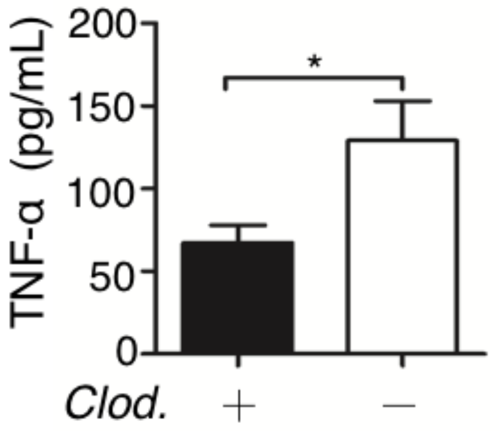

Fig. S9. Lupus IgG enhanced serum TNF-α in normal mice but not in macrophage-depleted mice. Mice were pretreated with *Clod. Liposomes* or vehicle on two consecutive days before SLE-IgG injection. The secretion of IFN-γ induced by SLE-IgG on the sections of B6 mice with or without anti-ASGM1 mAb pretreatment were examined by immunofluorescence 18 hours after SLE-IgG injection. (The result is representative of three independent experiments, n= 7 mice per group/experiment); *p<0.05

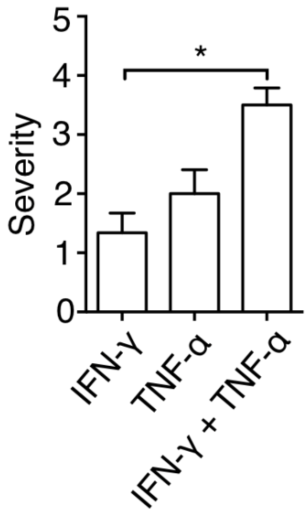

Fig. S10. Severity of liver inflammation 48h after intrahepatic injection of reconstituted TNF-α (200 ng/mouse) or/and IFN-γ (500 ng/mouse) in mice. *p<0.05, ***p<0.001 (The result is representative of three independent experiments, n= 5 mice per group/experiment); *p<0.05

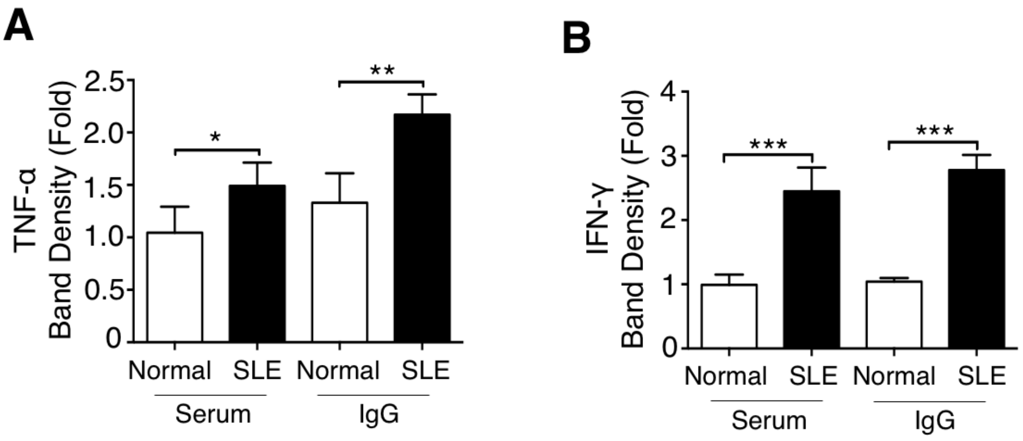

Fig. S11. C57BL/6 mice received serum/IgG from SLE patients or healthy individual administration as indicated. Total liver proteins were analyzed for hepatic inflammatory cytokine TNF-α (A) and IFN-γ (B) production by Western blotting using β-Actin as the normalization control. *p<0.05, **p<0.01, ***p<0.001 were considered statistically significant by analysis of variance for 6 mice per experimental group. Bands were quantified using ImageJ (Mac OS X, Ver. 1.47V; NIH, Bethesda, MD) and normalized to actin and the ratio was determined.

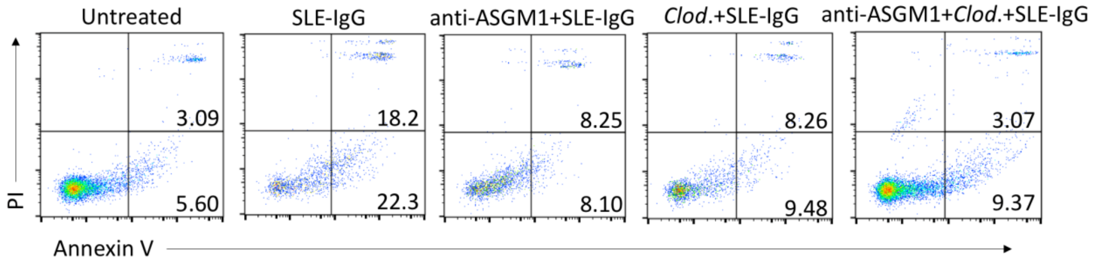

Fig. S12. Analysis of apoptotic hepatocytes 48h after intrahepatic injection of SLE-IgG in C57BL/6 mice with or without NK depletion using anti-ASGM1 antibody treatment, and the Annexin V *vs*. PI plots for those representative samples in Fig 4G.

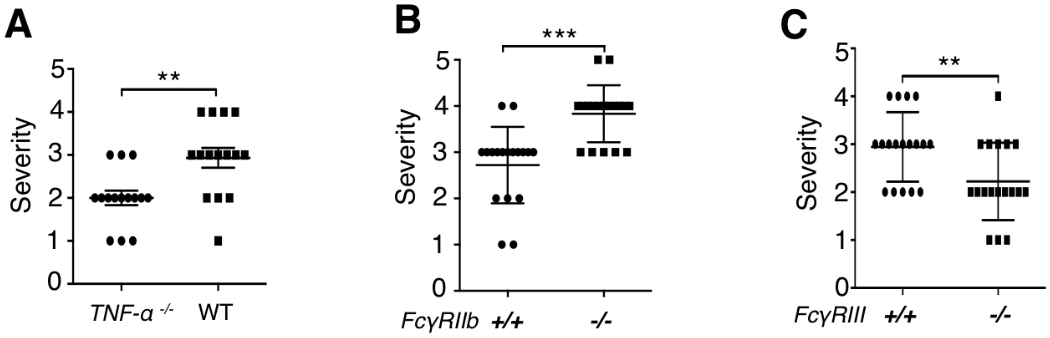

Fig. S13. We combined the data of all the three independent repeated experiments and made statistical analysis, Individual data points +/- SD of Figs 3D (n=15) middle panel and 5A (n=18) first and third panels are presenting here as (A), (B), and (C), respectively. **p<0.01, ***p<0.001
